# Biomarker analysis for patients with pancreatic cancer treated with nanoliposomal irinotecan plus 5-fluorouracil/leucovorin

**DOI:** 10.1186/s12885-023-10542-w

**Published:** 2023-01-20

**Authors:** Takeshi Kawakami, Akiko Todaka, Kotoe Oshima, Kunihiro Fushiki, Satoshi Hamauchi, Takahiro Tsushima, Tomoya Yokota, Yusuke Onozawa, Hirofumi Yasui, Kentaro Yamazaki

**Affiliations:** 1 Division of Gastrointestinal Oncology, Shizuoka Cancer Center, Shizuoka, Japan; 2 Division of Clinical Oncology, Shizuoka Cancer Center, Shizuoka, Japan

**Keywords:** Pancreatic cancer, Nanoliposomal irinotecan, GPS, NLR, Biomarker

## Abstract

**Background:**

Nanoliposomal irinotecan plus fluorouracil/leucovorin (5-FU/LV) is a standard second-line therapy for patients with pancreatic cancer. Identification of biomarkers is important to determine appropriate treatment strategies. We investigated the clinical practice outcomes and biomarkers associated with the nanoliposomal irinotecan plus 5-FU/LV regimen.

**Methods:**

We retrospectively reviewed the data of patients treated with nanoliposomal irinotecan plus 5-FU/LV as a second or subsequent treatment after gemcitabine-based therapy between June 2020 and March 2021 at Shizuoka Cancer Center.

**Results:**

We analyzed 55 consecutive patients who met the selection criteria. At a median of 9.4 months, median progression-free survival (PFS) and median overall survival (OS) were 2.3 and 6.6 months, respectively. Multivariate analysis showed that Glasgow prognostic score (GPS) was significantly associated with PFS (hazard ratio [HR] 2.16; 95% confidence interval [CI] 1.09–4.30; *P* = 0.028) and OS (0 vs. 1 or 2: HR 2.46; 95% CI 1.15–5.25; *P* = 0.029). The OS was significantly longer in patients with CA19–9 response than in those without CA19–9 response (12.6 vs. 5.6 months; HR 0.24; 95% CI 0.08–0.75; *P* = 0.014).

**Conclusions:**

Nanoliposomal irinotecan was efficacious and tolerable in clinical practice. GPS and CA19–9 response were good candidates as predictive biomarkers, whereas GPS was a good candidate prognostic biomarker for the nanoliposomal irinotecan plus 5-FU/LV regimen.

**Supplementary Information:**

The online version contains supplementary material available at 10.1186/s12885-023-10542-w.

## Background

Pancreatic cancer carries a poor prognosis and is the seventh leading cause of death worldwide [1] and the fourth largest cause of death in Japan [2]. Systemic chemotherapy is the standard treatment for locally advanced or metastatic pancreatic cancer. In Japan, FOLFIRINOX (FFX: oxaliplatin, irinotecan, plus fluorouracil/leucovorin [5-FU/LV]) or gemcitabine (GEM) plus nab-paclitaxel are recommended as first-line therapies. While there is no standard second-line chemotherapy, GEM plus nab-paclitaxel is commonly administered after the failure of FFX, and FOLFIRI (irinotecan plus 5-FU/LV), FOLFOX (oxaliplatin plus 5-FU/LV), or S-1 monotherapy is administered after the failure of GEM plus nab-paclitaxel as community standard treatments.

The NAPOLI-1 study demonstrated the superiority of nanoliposomal irinotecan in combination with 5-FU/LV with a hazard ratio of 0.67 (95% confidence interval [CI]: 0.49–0.76). This regimen was compared to 5-FU/LV monotherapy for patients with pancreatic cancer after demonstrating a refractory response to a GEM-containing regimen, with overall survival (OS) as the primary endpoint [3]. A randomized phase 2 trial of nanoliposomal irinotecan plus 5-FU/LV was performed to confirm the efficacy and feasibility of this regimen in Japanese patients [4]. As a primary endpoint, progression-free survival (PFS) was significantly longer in patients who received nanoliposomal irinotecan plus 5-FU/LV compared to those who received 5-FU/LV, with a hazard ratio of 0.60 (95% CI 0.37–0.98). The safety profile was similar to that of the NAPOLI-1 study. The combination therapy of nanoliposomal irinotecan plus 5-FU/LV was approved in Japan in 2019. Although this regimen is now recommended as second-line therapy after failure of the GEM-containing regimen, the efficacy and safety data of nanoliposomal irinotecan plus 5-FU/LV require elucidation in clinical practice settings.

The efficacy of nanoliposomal irinotecan is considered to be limited in patients previously treated with irinotecan. Subgroup analysis of the NAPOLI-1 study revealed that nanoliposomal irinotecan did not have additional benefit in patients who received irinotecan [5]. Moreover, the OS was significantly shorter in patients refractory to irinotecan than in those with nonrefractory response [6]. Several reports evaluated the efficacy of nanoliposomal irinotecan in patients previously treated with irinotecan in Japanese clinical practice.

Several prognostic biomarkers, including neutrophil-to-lymphocyte ratio (NLR) and Glasgow prognostic score (GPS) as inflammatory markers have been reported for patients with pancreatic cancer undergoing chemotherapy [7–9]. Subgroup analysis of the NAPOLI-1 study found that age ≤ 65 years, Karnofsky performance status score ≥ 90, NLR ≤ 5, CA19–9 <  59 × upper limit of normal, and fewer liver metastases were associated with longer OS [10]. Therefore, NLR is considered as a useful inflammatory marker for patients receiving nanoliposomal irinotecan plus 5-FU/LV. GPS, a cumulative score based on C-reactive protein and albumin, was originally developed by Forrest LM et al. [11]. GPS and NLR were used as prognostic biomarkers in several studies, including studies of patients with pancreatic cancer [8, 12, 13]. However, questions remain as to whether GPS or NLR is more useful. The CA19–9 response has been used as a predictive biomarker in recent clinical trials. In exploratory analyses of the ACCORD11/PRODIGE4 and MPACT trials, patients who demonstrated a CA19–9 response during treatment exhibited significantly longer survival than those who did not [14, 15].

Identification of biomarkers is important to determine appropriate treatment strategies. Therefore, we investigated the efficacy and safety of nanoliposomal irinotecan plus 5-FU/LV in clinical practice and explored predictive and prognostic biomarkers for patient outcomes.

## Methods

### Patients and methods

We retrospectively investigated patients treated with nanoliposomal irinotecan plus 5-FU/LV as second or later line treatment after GEM-based therapy between Jun 2020 and Mar 2021 at Shizuoka Cancer Center. We collected data from electric medical records. Selection criteria were age > 20 years at the initiation of nanoliposomal irinotecan plus 5-FU/LV treatment, Eastern Cooperative Oncology Group performance status (ECOG PS) 0–2, clinically diagnosed unresectable locally advanced or metastatic status, refractory to or intolerant of GEM-containing regimen, and adequate organ 5-FUnctions. We excluded patients with neuroendocrine carcinoma and other advanced solid tumors. The tumor response was evaluated based on RECIST version 1.1 criteria. Adverse events were evaluated using CTCAE version 5.0.

We also evaluated the NLR and CA19–9 responses. CA19–9 response was defined as > 50% decrease in CA19–9 from baseline during nanoliposomal irinotecan plus 5-FU/LV treatment. Cutoff NLR values differ across studies [7, 16, 17]. In two phase 3 studies, an NLR cutoff value of 5 was used for subgroup analysis [9, 10]. Therefore, we used an NLR cutoff value of 5 in the present study. GPS was evaluated by combining CRP and albumin (Alb): CRP ≤ 1.00 mg/dL and Alb ≥3.5 g/dL = GPS 0, CRP > 1.00 mg/dL or Alb < 3.5 g/dL = GPS 1, and CRP > 1.00 mg/dL and Alb < 3.5 g/dL = GPS 2. This study was approved by the Ethics Committee of Shizuoka Cancer Center (approval number 2933). All methods were conducted in accordance with the Declaration of Helsinki (1964 and later versions). Written informed consent was obtained from all patients.

### Treatment

The initial, biweekly dose of nanoliposomal irinotecan was 70 mg/m^2^ for *UGT1A1* wild-type or 50 mg/m^2^ for *UGT1A1* homozygous or double-heterotype. The fluorouracil dose was 2400 mg/m^2^ and the leucovorin dose was 250 mg/m^2^. The course of nanoliposomal irinotecan plus 5-FU/LV was repeated every two weeks until disease progression, unacceptable toxicity, or patient refusal. The nanoliposomal irinotecan dose was reduced to 50 mg/m^2^ or 43 mg/m^2^ and 5-FU to 1800 or 1350 mg/m^2^, depending on toxicity or at the physician’s discretion. All patients were administered palonosetron and dexamethasone as antiemetic agents.

### Statistical analyses

OS was defined as the duration between the date of initiation of nal-IRI plus 5-FU/LV and death, the date of the last follow-up, or the study’s cutoff date (September 30, 2021). PFS was defined as the duration between the date of initiation of nal-IRI plus 5-FU/LV and the date of confirmation of disease progression, the last follow-up, or the study’s cutoff date. OS and PFS were estimated using the Kaplan–Meier method and were compared using the log-rank test. Multivariate Cox regression analyses were performed to estimate the effect of several factors (ECOG PS, age, NLR, GPS) on survival. The analyses were performed using the statistical software R (R Foundation for Statistical Computing, v. 4.0.2). All *P*-values were two-sided, and *P* < 0.05 was considered statistically significant.

## Results

### Patients’ backgrounds

Fifty-five patients were selected for this study; their characteristics are shown in Table 1. The median age (range) was 67.5 (53–83) years. The most common histology type was adenocarcinoma, and homo- or double-heterotype *UGT1A1* statuses were observed in nine patients. As for disease status, three patients had unresectable locally advanced (UR-LA) disease, and the remaining had metastatic disease. Nine patients were previously treated with irinotecan. Median CA19–9 (range) was 315 (2–18,388) U/mL. Thirteen patients had >NLR 5 and 12 patients were GPS 2.

**Table 1:** Patients’ characteristics

*N* = 55		
Age	Median (range), years	67 (53–83)
Sex	Male/female	36/19
ECOG PS	0/1/2	14/37/4
Pathology	Adeno/adenosquamous/UN^1^	49/3/3
MSI status	MSI-H/MSS/UN^1^	0/34/21
*UGT1A1* status	WT^2^/single hetero/homo/double hetero/UN^1^	19/26/6/3/1
Location	Head/body/tail	23/18/14
Disease status	UR-LA^3^ / M^4^	3/52
Metastatic site	HEP/PER/LYM/PUL	39/18/18/16
Number of metastatic sites	0/1/≥2	3/25/27
Baseline CA19–9	Median (range), U/mL	315 (2–18,388)
Prior irinotecan	Yes	9
Number of previous courses of palliative chemotherapy	1/2/3	25/28/2
NLR^5^	< 5/ ≥ 5	42/13
GPS^6^	0/1/2	29/14/12

^1^unknown, ^2^wild type, ^3^unresectable-locally advanced, ^4^metastatic, ^5^ neutrophile-to-lymphocyte ratio, ^6^Glasgow prognostic score

### Efficacy

**Fig. 1:**
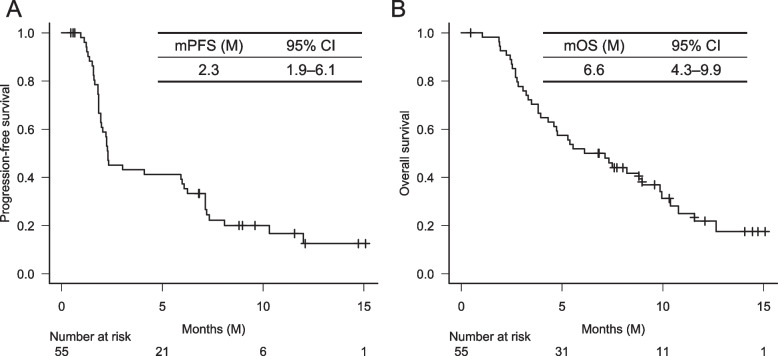
Progression-free survival (A) and overall survival (B) in patients treated with nanoliposomal irinotecan plus 5-FU/LV (fluorouracil/leucovorin)

**Fig. 2:**
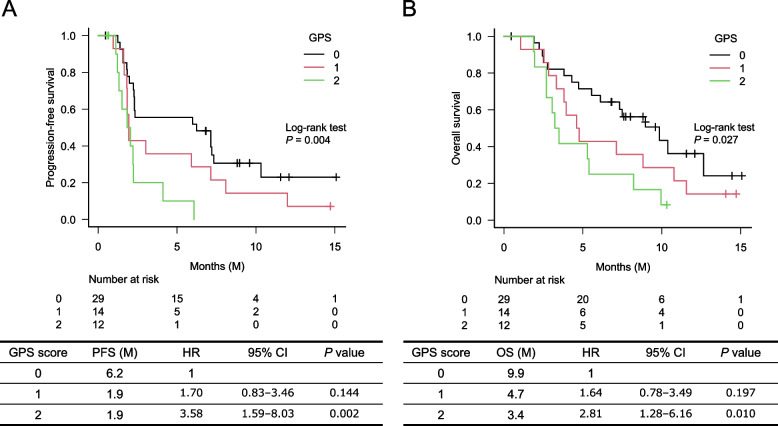
PFS (A) and OS (B) according to Glasgow Prognostic Score (GPS) score

At the study cutoff on September 30, 2021, the median observation time was 9.4 (0.5–14.9+) months. The median PFS and OS were 2.3 (95% CI 1.9–6.1) and 6.6 (95% CI 4.3–9.9) months, respectively (Fig. 1A, B). The response rate of 45 patients with measurable lesions was 11.1%, and the disease control rate was 35.6%. PFS and OS, according to GPS, were significantly different among the 3 groups; GPS 0 indicated the best prognosis and 2 the worst prognosis (Fig. 2A, B). The median PFS and OS of patients treated with and without prior irinotecan were 1.9 months and 2.3 months (*P* = 0.364), and 4.7 months and 8.2 months (*P* = 0.225), respectively. None of the seven patients previously treated with irinotecan were responders, and PFS and OS tended to be shorter in these patients (Supplementary Fig. 1A, 1B).

### Adverse events

Treatment-emergent adverse events are shown in Table 2. Adverse events of grade 3 or higher observed in > 10%, were neutropenia (11%) and anorexia (13%). No febrile neutropenia or treatment-related deaths were observed.

**Table 2:** Treatment-emergent adverse events*

	Any grade		≥Grade 3	
	N	(%)	N	(%)
Neutropenia	30	(54)	6	(11)
Anemia	21	(38)	1	(2)
Thrombocytopenia	6	(11)	1	(2)
Nausea	33	(59)	4	(7)
Vomiting	4	(7)	1	(2)
Anorexia	39	(70)	7	(13)
Diarrhea	8	(14)	1	(2)
Fatigue	34	(61)	3	(5)
Stomatitis	5	(9)	0	(0)
Febrile neutropenia	0	(0)	0	(0)

^*^No treatment-related death was observed

### Treatment exposure

Treatment exposures are shown in Table 3. The median treatment course (range) was 5 (1–30+), and eight patients continued nanoliposomal irinotecan plus 5-FU/LV at the study cutoff. The main reasons for discontinuation were progressive disease (*n* = 42), allergic response to the regimen (*n* = 3), congestive heart failure (*n* = 1), and cholinergic syndrome uncontrolled by atropine (n = 1); one patient underwent conversion surgery. Initial dose reductions of nanoliposomal irinotecan or 5-FU were performed in 31 patients because of unknown *UGT1A1* status at initial administration (*n* = 8), age (*n* = 10), and poor PS (n = 10). Of the six patients whose *UGT1A1* status was initially unknown and whose *UGT1A1* status was proven to be wild-type or single heterozygous, one patient had their dose of nanoliposomal irinotecan increased by one level. Eighteen patients had their doses reduced after the first course due to fatigue (*n* = 10), nausea (*n* = 6), and anorexia (n = 6). Twenty-two patients received subsequent chemotherapies, including FOLFOX (*n* = 20), S-1 monotherapy (n = 2), GEM plus nab-paclitaxel (n = 1), and FOLFIRI (n = 1).

**Table 3:** Treatment exposure

	*N* (%)	
On treatment	8	(14)
Discontinuation	47	(85)
Refractory	42	(76)
Intolerant	3	(5)
Conversion surgery	1	(2)
Initial dose reduction either nanoliposomal irinotecan or 5-FU	31	(56)
*UGT1A1* not available at the initial administration	8*	(15)
Age	10	(18)
PS	3	(5)
Others	10	(18)
Dose reduction after one dose	18	(33)
Reasons for dose reduction		
Fatigue	10	(18)
Nausea	6	(11)
Anorexia	6	(11)
Diarrhea	2	(4)
Others	2	(4)
Post-treatment^†^	22	(40)
FOLFOX	20	(36)
S-1	2	(4)
GEM + nab-PTX	1	(2)
FOLFIRI	1	(2)

*Of the 6 patients with wild-type or single heterozygous *UGT1A1*, nanoliposomal irinotecan dose was increased in only one patient. ^†^ Duplicate cases were present

### Prognostic and predictive factors for nanoliposomal irinotecan

The results of the univariate and multivariate analysis for PFS are shown in Table 4. Among possible predictive factors for PFS (age, ECOG PS, liver metastasis, prior treatment with irinotecan, NLR, and GPS), multivariate analysis using the Cox-hazard model revealed that NLR (≤5 vs. > 5: HR 2.26; 95% CI 1.09–6.53; *P* = 0.032) and GPS (0 vs. 1 or 2: HR 2.16; 95% CI 1.09–4.30; *P* = 0.028) were independent predictors (Table 4). The univariate and multivariate analysis for OS defined the prior irinotecan (− vs. +: HR 1.62; 95% CI 1.13–7.21; *P* = 0.027) and GPS (0 vs. 1 or 2: HR 2.46; 95% CI 1.15–5.25; *P* = 0.029) were independent prognostic factors; however, NLR was not (≤5 vs. > 5: HR 1.51; 95% CI 0.64–3.61; *P* = 0.349) (Table 5).

**Table 4:** Univariate and multivariate analysis for progression-free survival

Univariate analysis					Multivariate analysis		
Variables		HR	95% CI	*P* value	HR	95% CI	P value
Age (years)	≤65 vs. > 65	0.81	0.42–1.55	0.521			
ECOG PS	0–1 vs. 2	1.13	0.35–3.69	0.837			
Liver metastasis	− vs. +	1.17	0.60–2.29	0.652			
Prior irinotecan	− vs. +	1.45	0.64–3.29	0.372	2.33	0.92–5.91	0.075
NLR	≤5 vs. > 5	2.26	1.10–4.67	0.027	2.67	1.09–6.53	0.032
GPS	0 vs. 1–2	2.17	1.17–4.02	0.014	2.16	1.09–4.30	0.028

**Table 5:** Univariate and multivariate analysis for overall survival

Valuables		Univariate analysis			Multivariate analysis		
		HR	95% CI	P value	HR	95% CI	P value
Age (years)	≤65 vs. > 65	0.81	0.42–1.56	0.522			
ECOG PS	0–1 vs. 2	1.38	0.42–4.50	0.596			
Liver metastasis	− vs. +	1.29	0.61–2.73	0.501			
Prior irinotecan	− vs. +	1.62	0.74–3.54	0.230	2.85	1.13–7.21	0.027
NLR	≤5 vs. > 5	1.76	0.87–3.55	0.117	1.51	0.64–3.61	0.349
GPS	0 vs. 1–2	2.04	1.08–3.89	0.029	2.46	1.15–5.25	0.029

**Fig. 3:**
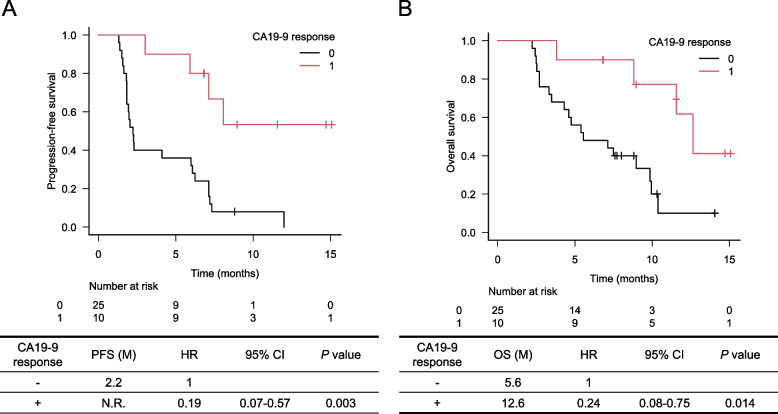
PFS (A) and OS (B) according to CA19–9 response

Of the 36 patients with elevated CA19–9 at baseline, 26 experienced a CA19–9 response. PFS was significantly longer in CA19–9 responders than in nonresponders (not reached vs. 2.2 months; HR 0.19; 95% CI 0.07–0.57; *P* = 0.003) (Fig. 3A). OS was also significantly longer in CA19–9 responders than nonresponders (12.6 vs. 5.6 months; HR 0.24; 95% CI 0.08–0.75; *P* = 0.014) (Fig. 3B).

## Discussion

We found OS for nanoliposomal irinotecan plus 5-FU/LV combination therapy for pancreatic cancer refractory to GEM-based chemotherapy to be comparable to that of the Japanese phase II study [4] and NAPOLI-1 study [3]. However, our patients’ PFS was generally worse than those observed in the NAPOLI-1 study. It is probably because we included patients who initiated therapy despite having a poor disease status while waiting for approval of nanoliposomal irinotecan. We also included patients treated with FOLFIRI who were switched to nanoliposomal irinotecan plus 5-FU/LV after approval of nanoliposomal irinotecan. Therefore, we likely undervalued the anti-tumor effect of nanoliposomal irinotecan plus 5-FU/LV for these patients.

The efficacy of nanoliposomal irinotecan after the failure of an irinotecan-containing regimen, such as FFX, is an important clinical question. In the present study, OS tended to be shorter in patients with prior exposure to irinotecan than in those without prior exposure to irinotecan. The active metabolite of nanoliposomal irinotecan is SN-38, as is irinotecan, suggesting that it is difficult to restore the anti-tumor effect of irinotecan only by improving drug delivery. Multivariate analysis showed that history of prior irinotecan administration was an independent prognostic factor for OS in patients receiving nanoliposomal irinotecan, suggesting that nanoliposomal irinotecan might not be considered in patients who are refractory to irinotecan.

We observed more incidences of grade 3 or higher neutropenia, nausea, and anorexia than in previous reports [3, 4]. It is probably because our patients were heavily treated, and some may have had cancer cachexia. However, almost all patients who experienced severe adverse events continued the nanoliposomal irinotecan plus 5-FU/LV regimen after appropriate dose modification. Although the careful patient selection and appropriate management are necessary, this regimen was generally well-tolerated.

Inflammatory markers such as GPS and NLR are prognostic factors for unresectable pancreatic cancer [9, 10]. NLR was predictive for PFS but not OS. A cutoff value of NLR 5 may be inappropriate for patients who receive second-line treatment. Since there is no settled cutoff value for NLR, we used a cutoff value of 5 was used in this study based on previous reports [9, 10]. Because our study cohort was relatively small, our results should be examined in future, well-powered studies. A retrospective study comparing NLR to inflammatory markers such as GPS showed GPS as the most reliable predictive biomarker. Our results indicated that the GPS was a stronger predictor than NLR.

Patients with GPS 2 had the worst OS and PFS. These patients demonstrated a poor prognosis and were more likely to experience early progression; therefore, patients with other GPS scores should be carefully evaluated to determine the indication for treatment. Future trials may consider using GPS as a stratification factor for second or subsequent pancreatic cancer treatments.

The CA19–9 response has been adopted by several clinical pancreatic cancer trials as an on-treatment marker. Since the survival time of CA19–9 responders was significantly better than nonresponders in this study, we consider the CA19–9 response to be an on-treatment predictive biomarker. CA19–9 response may be useful for determining if nanoliposomal irinotecan plus 5-FU/LV and intensified supporting care should be continued in patients who experience severe adverse events. We expect that patients with a CA 19–9 response during nanoliposomal irinotecan plus 5-FU/LV will enjoy better long-term survival. Therefore, if a CA19–9 response is observed, physicians should consider increasing supportive care to maintain the dose intensity and avoid intolerance to maximize the regimen’s anti-tumor effects. However, the CA19–9 response cannot be used in patients without elevated CA19–9 at chemotherapy initiation, or in patients with a negative Lewis-type blood group.

This study has several limitations. First, selection bias was inevitable since this was a monocentric retrospective study. Moreover, our sample size is small and requires validation using an external cohort. However, we identified GPS and the CA19–9 response as biomarkers that may help inform treatment decision-making in clinical practice settings.

## Conclusions

Nanoliposomal irinotecan was efficacious and well-tolerated in a clinical practice setting. Careful patient selection and appropriate management are necessary when administering this regimen because of the relatively high frequency of grade 3 or higher neutropenia and anorexia. GPS and history of prior irinotecan administration could be a good candidate prognostic biomarker for nanoliposomal irinotecan plus 5-FU/LV, and GPS could be used as a stratification factor in future clinical trials for patients with pancreatic cancer receiving second-line treatment.

## Supplementary Information

**Additional file 1.** Supplementary Fig. 1. PFS (A) and OS (B) of patients with or without a refractory response to prior nonliposomal irinotecan.

## Data Availability

The datasets generated during and/or analyzed during the current study are available from the corresponding author upon reasonable request.

## Abbreviations

ECOG: Eastern Cooperative Oncology Group
FFX: FOLFIRINOX (oxaliplatin, irinotecan, plus fluorouracil/leucovorin [5-FU/LV])
GEM: gemcitabine
GPS: Glasgow Prognostic Score
NLR: neutrophil-to-lymphocyte ratio
OS: overall survival
PFS: progression-free survival

## References

[1] Estimated number of deaths in 2020, world, females, all ages. GLOBOCAN. 2020. https://gco.iarc.fr/today/online-analysis-pie?v=2020&mode=cancer&mode_population=continents&population=900&populations=900&key=total&sex=0&cancer=39&type=1&statistic=5&prevalence=0&population_group=0&ages_group%5B%5D=0&ages_group%5B%5D=17&nb_items=7&group_cancer=1&include_nmsc=1&include_nmsc_other=1&half_pie=0&donut=0. Accessed 14 Sep 2022.

[2] Latest cancer statistics. Ministry of Labor, health and welfare, Japan. 2022. https://ganjoho.jp/reg_stat/statistics/stat/summary.html. Accessed 14 Sep 2022.

[3] Wang-Gillam A, Li CP, Bodoky G, Dean A, Shan YS, Jameson G (2016). Nanoliposomal irinotecan with fluorouracil and folinic acid in metastatic pancreatic cancer after previous gemcitabine-based therapy (NAPOLI-1): a global, randomised, open-label, phase 3 trial. Lancet..

[4] Ueno M, Nakamori S, Sugimori K, Kanai M, Ikeda M, Ozaka M (2020). Nal-IRI + 5-FU/LV versus 5-FU/LV in post-gemcitabine metastatic pancreatic cancer: randomized phase 2 trial in Japanese patients. Cancer Med.

[5] Macarulla Mercadé T, Chen LT, Li CP, Siveke JT, Cunningham D, Bodoky G (2020). Liposomal irinotecan + 5-FU/LV in metastatic pancreatic cancer: subgroup analyses of patient, tumor, and previous treatment characteristics in the pivotal NAPOLI-1 trial. Pancreas..

[6] Smith CJ, Bekaii-Saab TS, Cook KD, Eiring RA, Halfdanarson TR, Hanna M (2021). Nanoliposomal irinotecan (Nal-IRI)-based chemotherapy after irinotecan -based chemotherapy in patients with pancreas cancer. Pancreatology..

[7] Iwai N, Okuda T, Sakagami J, Harada T, Ohara T, Taniguchi M (2020). Neutrophil to lymphocyte ratio predicts prognosis in unresec` pancreatic cancer. Sci Rep.

[8] Yamada S, Fujii T, Yabusaki N, Murotani K, Iwata N, Kanda M (2016). Clinical implication of inflammation-based prognostic score in pancreatic cancer: Glasgow prognostic score is the most reliable parameter. Med..

[9] Goldstein D, El-Maraghi RH, Hammel P, Heinemann V, Kunzmann V, Sastre J (2015). Nab-paclitaxel plus gemcitabine for metastatic pancreatic cancer: long-term survival from a phase III trial. J Natl Cancer Inst.

[10] Wang-Gillam A, Hubner RA, Siveke JT, Von Hoff DD, Belanger B, de Jong FA (2019). NAPOLI-1 phase 3 study of liposomal irinotecan in metastatic pancreatic cancer: final overall survival analysis and characteristics of long-term survivors. Eur J Cancer.

[11] Forrest LM, McMillan DC, McArdle CS, Angerson WJ, Dunlop DJ (2003). Evaluation of cumulative prognostic scores based on the systemic inflammatory response in patients with inoperable non-small-cell lung cancer. Br J Cancer.

[12] Mcmillan DC (2013). The systemic inflammation-based Glasgow prognostic score: a decade of experience in patients with cancer. Cancer Treat Rev.

[13] Forrest LM, McMillan DC, McArdle CS, Angerson WJ, Dunlop DJ (2004). Comparison of an inflammation-based prognostic score (GPS) with performance status (ECOG) in patients receiving platinum-based chemotherapy for inoperable non-small-cell lung cancer. Br J Cancer.

[14] Robert M, Jarlier M, Gourgou S, Desseigne F, Ychou M, Bouché O (2017). Retrospective analysis of CA19-9 decrease in patients with metastatic pancreatic carcinoma treated with folfirinox or gemcitabine in a randomized phase III study (ACCORD11/PRODIGE4). Oncology..

[15] Chiorean EG, von Hoff DD, Reni M, Arena FP, Infante JR, Bathini VG (2016). CA19-9 decrease at 8 weeks as a predictor of overall survival in a randomized phase III trial (MPACT) of weekly nab-paclitaxel plus gemcitabine versus gemcitabine alone in patients with metastatic pancreatic cancer. Ann Oncol.

[16] Yang JJ, Hu ZG, Shi WX, Deng T, He SQ, Yuan SG (2015). Prognostic significance of neutrophil to lymphocyte ratio in pancreatic cancer: a meta-analysis. World J Gastroenterol.

[17] Toledano-Fonseca M, Cano MT, Inga E, Gómez-España A, Guil-Luna S, García-Ortiz MV (2021). The combination of neutrophil–lymphocyte ratio and platelet–lymphocyte ratio with liquid biopsy biomarkers improves prognosis prediction in metastatic pancreatic cancer. Cancers..

