# Supplementary material for: Biomarker analysis for patients with pancreatic cancer treated with nanoliposomal irinotecan plus 5-fluorouracil/leucovorin
Source: BMC Cancer. 2023 Jan 20;23:68. doi: 10.1186/s12885-023-10542-w (PMC9854093; doi:10.1186/s12885-023-10542-w)
Supplement: Supplementary file 1 — Additional file 1. Supplementary Fig. 1. PFS (A) and OS (B) of patients with or without a refractory response to prior nonliposomal irinotecan. [file 12885_2023_10542_MOESM1_ESM.pptx]

## Slide 1
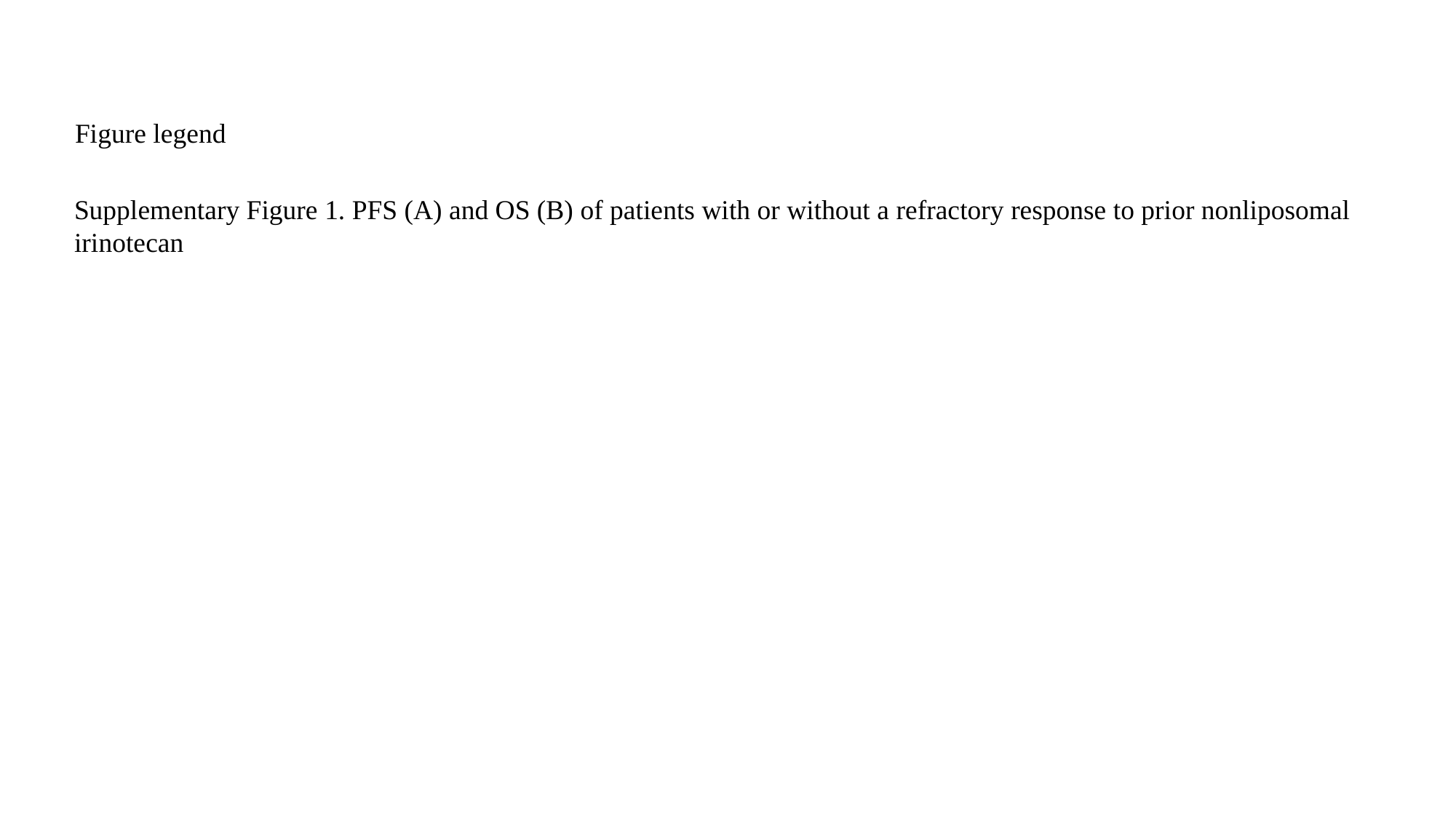

Figure legend
Supplementary Figure 1. PFS (A) and OS (B) of patients with or without a refractory response to prior nonliposomal irinotecan

## Slide 2
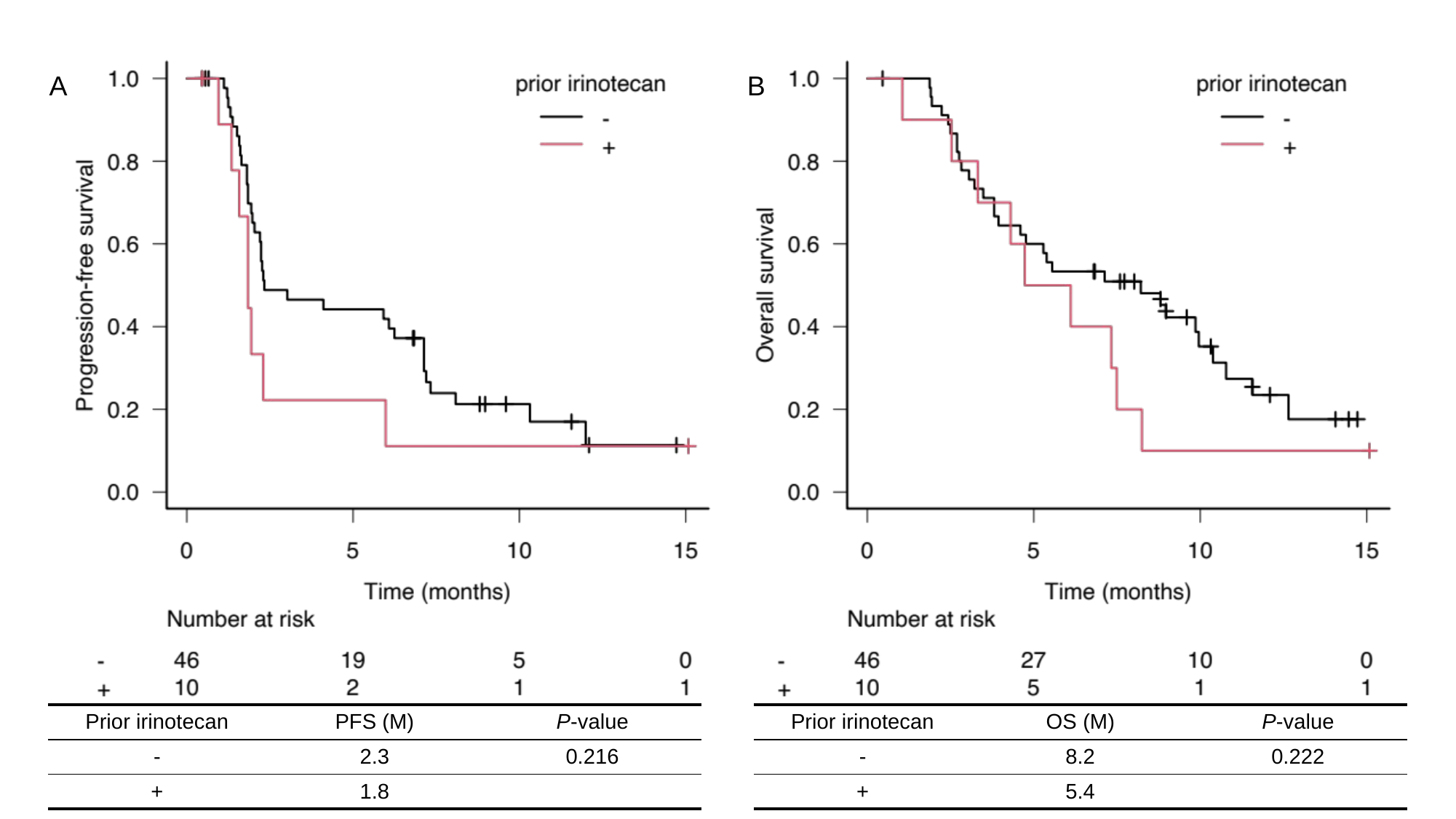

A
B
| Prior irinotecan | PFS (M) | P-value |
| --- | --- | --- |
| - | 2.3 | 0.216 |
| + | 1.8 | |
| Prior irinotecan | OS (M) | P-value |
| --- | --- | --- |
| - | 8.2 | 0.222 |
| + | 5.4 | |
